# A Lipoxygenase from Red Alga *Pyropia haitanensis*, a Unique Enzyme Catalyzing the Free Radical Reactions of Polyunsaturated Fatty Acids with Triple Ethylenic Bonds

**DOI:** 10.1371/journal.pone.0117351

**Published:** 2015-02-06

**Authors:** Zhujun Zhu, Feijian Qian, Rui Yang, Juanjuan Chen, Qijun Luo, Haimin Chen, Xiaojun Yan

**Affiliations:** 1 Key Laboratory of Applied Marine Biotechnology, Ningbo University, Ningbo, Zhejiang, 315211, China; 2 School of Marine Science, Ningbo University, Ningbo, Zhejiang, 315211, China; Stazione Zoologica Anton Dohrn, Naples, ITALY

## Abstract

Lipoxygenases (LOXs) are key enzymes to regulate the production of hormones and defensive metabolites in plants, animals and algae. In this research, a full length LOX gene has been cloned and expressed from the red alga *Pyropia haitanensis* (Bangiales, Rhodophyta) gametophyte (PhLOX2). Subsequent phylogenetic analysis showed that such LOX enzymes are separated at the early stage of evolution, establishing an independent branch. The LOX activity was investigated at the optimal pH of 8.0. It appears that PhLOX2 is a multifunctional enzyme featuring both lipoxygenase and hydroperoxidase activities. Additionally, PhLOX2 exhibits remarkable substrate and position flexibility, and it can catalyze an array of chemical reactions involving various polyunsaturated fatty acids, ranging from C18 to C22. As a matter of fact, mono-hydroperoxy, di-hydroperoxy and hydroxyl products have been obtained from such transformations, and eicosapentaenoic acid seem to be the most preferred substrate. It was found that at least triple ethylenic bonds are required for PhLOX2 to function as a LOX, and the resulting hydroxy products should be originated from the PhLOX2 mediated reduction of mono-hydroperoxides, in which the hydrogen abstraction occurs on the carbon atom between the second and third double bond. Most of the di-hydroperoxides observed seem to be missing their mono-position precursors. The substrate and position flexibility, as well as the function versatility of PhLOXs represent the ancient enzymatic pathway for organisms to control intracellular oxylipins.

## Introduction

Compounds derived from the oxidative metabolism of polyunsaturated fatty acids (PUFAs) are found in terrestrial plants, marine algae and animals, serving as an intrinsic response to external stimuli [1]. Products synthesized via such pathways are collectively called oxylipins, and the multi-step processes involved are often initiated by lipoxygenases (LOXs) [2]. In general, LOXs constitute a large gene family of nonheme iron containing fatty acid dioxygenases, which are ubiquitous in higher plants and animals [3]. In fact, they can serve as the catalyst for regio- and stereo-specific insertion of molecular oxygen into PUFAs containing one or more (*1Z*, *4Z*)-pentadiene system, e.g., linoleic acid (LA), *a*-linolenic acid (ALA), arachidonic acid (AA), and eicosapentaenoic acid (EPA), resulting in the formation of (*2E*, *4Z*)-hydroperoxy diene fragment [4].

The biosynthesis of oxylipins is different for higher plants, mammals and algae. For instance, C18 PUFAs are the most common substrates for LOX enzymes in higher plants [5], while C20 PUFAs are predominant substrates for LOXs in mammals and invertebrate animals [3]. The activity of LOXs can be classified according to the positional specificity of oxygenation for PUFAs. In higher plants, the catalytic insertion of oxygen either takes place at the carbon atom C-9 or C-13 of the hydrocarbon backbone, which leads to the formation of 9-hydroperoxyoctadecatrienoic acid (HpOTE) or 13-HpOTE [6]. In mammals, however, AA can be oxygenated by LOXs at six different positions: C-5, C-8, C-9, C-11, C-12 or C-15 [7]. Marine algae live in a complex seawater environment. Like higher plants and mammals, algae can also produce variety of oxylipins served as chemical defense signals [8]. Based on the oxylipin structures identified so far, it was determined that marine algae probably contain 5-, 8-, 9-, 12-, and 15-LOXs that can act on eicosanoic (C20) PUFAs, as well as *ω*3-, *ω*6-, *ω*9-, and *ω*10-LOXs that can act on octadecanoids (C18) [9]. However, till now, very limited information is available on the enzymes that are responsible for the biosynthesis of those compounds, but only two genes encoding LOX have been identified in *Chondrus crispus*, which is surprising considering the diverse oxylipins observed in mairne algae [10].

*Pyropia haitanensis* is one of the commercially important macroalgae on the south-east coast of China. It was cultured in intertidal regions which are rapidly changing physical conditions due to the turning tides, thus, *P*. *haitanensis* has high levels of tolerance to various abiotic stressors. In has been reported that the lipid metabolic defense pathway of *P*. *haitanensis* seems to involve both C18 and C20 metabolism pathways when elicited by agaro-oligosaccharides, and the latter plays a leading role in the metabolic defense process [11]. However, no information is available about the LOX enzymes responsible for synthesis of oxylipins in *P*. *haitanensis*. We have obtained the full sequences of two LOX genes from *P*. *haitanensis* (PhLOX and PhLOX2). PhLOX is still under investigation. In this study, we reported the identification, cloning, heterologous expression, and functional characterization of PhLOX2 from *P*. *haitanenresis*. The multiple enzymatic activities of PhLOX2 raise interesting questions about the role of this enzyme in the formation of diverse oxylipins.

## Material and Methods

### Algae material

The 5–10 cm long *P*. *haitanensis* thalli were collected from the low intertidal zones along the coast of Hepu, Xiangshan, Ningbo, China (29°09’18”N, 121°54’05”W) in 2012. The location was authorized by Council of Hepu Town, Xiangshan County. We obtained permission to conduct the field studies by local government and the local department of fisheries. Samples were first dried in the shade, and then stored at -20°C. Before use, all samples were manually brushed, and then rinsed with filtered seawater to remove visible epiphytic foreign matters. The resulting materials were subsequently cleaned with 0.7% KI (W/V) for 15 min, followed by maintaining in autoclaved water in glass aquaria (40 μmol m^-2^ s^-1^, LD cycle 12:12 h) at 18–20°C for 24 h.

### Full-length cDNA clone and sequence analysis of *PhLOX2*

Coding regions of *PhLOX*2 gene have been partially identified by searching for cDNA clones showing homology with *Porphyra purpurea* lipoxygenase gene in the transcriptome database of *P*. *haitanensis* gametophyte (undisclosed data). Total RNA was first extracted from the *P*. *haitanensis* samples using the TaKaRa RNAiso Plus Reagent (TaKaRa, Dalian, China), and then it was reversely transcribed with the MMLV reverse transcriptase (TaKaRa, Dalian, China) from an anchored oligo-dT primer using the standard methods. For the isolation of *PhLOX*2 partial cDNA clones, two specific primers, *PhLOX2-F* (5′-GCTGACGATGCCCAAGTACTG-3′) and *PhLOX2-R* (5′-GCTGCTGTTGTTGGGTTCCT-3′), were designed for polymerase chain reaction (PCR) amplification with the 2 × HotStart Taq PCR MasterMix (BioTeke, Beijing, China). A DNA fragment with the desired size was generated, subcloned into the vector pMD-18T (TaKaRa, Dalian, China), and sequenced.

Full-length cDNAs of *PhLOX2* gene was isolated using the 5’- and 3’-RACE method (SMART RACE cDNA Amplification Kit, Clontech, Japan) with the following nest primers: *LOX2–5R1/LOX2–5R2*: 5′-TGATGCCAATCGTGTCCTGGAAGTG-3′/ 5′-AGGGACTTGCCCAGCGAAAAGAGGAGC-3′; *LOX2–3F1/LOX2–3F2*: 5′-GCGGTCGTCTCCATCATCTTCC-3′/5′-TCTTCCAGACGCTCTTTGCGCAC-3′.

Phylogenetic tree analysis was performed on the amino acid sequences of the selected LOXs and PhLOX2 using Clustalx, and the data collected were edited with TreeView and CorelDRAW 12 software. Sequences of LOXs have been obtained from higher plants, red algae, mammals, and prokaryotes.

### Expression and purification of PhLOX2

The corresponding ORF of the *PhLOX2* gene was amplified by the TaKaRa Ex Taq polymerase (TAKARA, Dalian, China) using special primers associated with the restriction enzyme site. The pairs of specific primers are described as follows, *PhLOX2-ORF-F*: 5′-GGAATTCCATATGATGGCGCACACCG-3′ (*Nde* I-site underlined) and *PhLOX2-ORF-R*: 5′-CCCAAGCTTCTAAATGTCAATCGACAGGG-3′ (Hind III-site underlined). The resulting 2.7-kb PCR product was first digested and ligated onto the Nde I and Hind III-digested expression vector pET-28a, and then transformed into *E*. *coli* BL21 cells to express a recombined protein, leading to the introduction of *N*-terminal his-tag. The cells were cultured in 400 ml medium at 37°C for 12 h. Next, the transformants were induced in the presence of 0.1 mM isopropyl thio-*β*-galactoside at 20°C for 16–24 h, and the resulting cells were then harvested and lysed. After centrifugation at 15,800 × g for 15 min, the supernatant was purified by a Ni-agarose column (6 x His-Tagged Protein Purification Kit, Cwbio, Beijing, China) to afford PhLOX2. The purity of PhLOX2 protein was evaluated by 10% SDS-PAGE electrophoresis. The gel analysis was performed with ImageJ program. The secondary structure predication was performed by Network Protein Sequence Analysis (http://npsa-pbil.ibcp.fr/cgi-bin/npsa_automat.pl?page=/NPSA/npsa_hnn.html). Size exclusion chromatography was performed on a Superdex 200 gel filtration column (GE-Healthcare, USA) eluted with 20 mM Tris-HCl buffer (pH 8.0) containing 200 mM NaCl at a flow rate of 0.5 ml min^-1^ on an AKTA prime plus chromatography system.

### LC-MS analysis

Products (hydroperoxy fatty acids and hydroxy fatty acids) obtained from the PhLOX2 catalyzed reactions were analyzed on a Finnigan Surveyor and TSQ Quantum Access analysis system (Thermo Fisher Scientific, USA) equipped with a high-resolution mass spectrometry. A Hypersil Gold C_18_ analytical column (100 mm × 2.1 mm, 3 μm, Thermo Fisher Scientific, USA) was employed, and all experiments were carried out at 30°C using a solvent system of acetonitrile (A)-0.2% acetic acid (B) with a flow rate of 0.2 ml min^-1^. Eluting was developed from 30% A to 55% A in 4 min, then to 80% A in 32 min, finally to 100% A in 4 min.

High-resolution mass spectrometry was performed on a Q Exactive hybrid quadrupole-Orbitrap mass spectrometer operating in the data dependent mode to automatically switch between full scan MS and MS/MS acquisition in negative ion mode. Survey full scan MS spectra with mass range scanning from 50–500 was acquired in the Orbitrap with 70,000 resolution (m/z 200) after accumulation of ions to a 1×10^6^ target value based on predictive AGC from previous full scan. Dynamic exclusion was set to 70 s. The maximum ion time is 250 ms. The MS/MS parameters were set as follows: AGC target 2×10^5^; maximum ion time 250 ms; isolation width 2 Da. Typical mass spectrometric conditions were: spray voltage, 2 kV; no sheath and auxiliary gas flow; heated capillary temperature, 275; normalized collision energy was set to 25%, and activation time to 20 ms.

### Substrate specificities of PhLOX2

The substrate specificity and catalytic reaction pathway of PhLOX2 have been determined by LC-MS analysis, using substrates including LA, ALA, *γ*-linolenic acid (GLA), AA, EPA, docosahexaenoic acid (DHA) (Cayman Chemical Company, Ann Arbor, MI, USA) and hydroperoxide intermediates 8-hydroperoxy eicosapentaenoic acid (HpEPE) and 7-hydroperoxy docosahexaenoic acid (HpDHE). The reaction was initiated by adding 0.5 μl 100 mM of substrate into a final volume of 500 μl 12 μM pure enzyme at 20°C. After 30 min incubation, the reaction was terminated, and the resulting mixture was extracted with 1 ml of ethyl acetate for 30 min at 4°C (980 × g). Subsequently, the extract was centrifuged at 14,600 × g at 4°C for 15 min. The upper phase was collected, dried under nitrogen, and re-dissolved in 500 μl of methanol for LC-MS analysis. For the control group, only 500 μl of buffer (pH 8.0) was added.

EPA and DHA were reacted with the purified enzyme at 20°C for 2 h and the ethyl acetate extract was separated by HPLC using an uBondapak C18 column (3.9×300 mm, Water Co.) to obtain 8-HpEPE and 7-HpDHE. The purified 8-HpEPE and 7-HpDHE were used for catalytic reaction.

In order to compare the substrate selectivity of PhLOX2, a sample was prepared by adding the mixture of four substrates (GLA, AA, EPA and DHA, each of them with a final concentration of 100 μM) into the enzyme solution for 30 min. To quantify the substrates utilization, six PUFAs standards obtained from Sigma Co. were solved in 30% acetonitrile to give a concentration of 100 μM. Working solutions were obtained by dilution of the solution with the 30% acetonitrile for LC-MS analysis. The calibration curves of six PUFAs standards were established by integrating the peak areas of different concentration of fatty acid standard. The analysis of substrate utilization is based on calibration curves.

### Detection of pH dependence and time course of PhLOX2 activities

To determine the “optimal” pH for PhLOX2, the enzyme assay was performed under different pH conditions, ranging from pH 5 to 10 (pH 5–7, 50mM phosphate buffer; pH8–10, 50mM Tris-HCl buffer). In 96-well plates, 100 mM GLA, AA, EPA or DHA was added until a final volume of 500 μl was achieved. Moreover, the enzyme activity was determined by monitoring the UV absorbance of hydroxy and hydroperoxy products at 235 nm (conjugated diene system of the hydroxy fatty acids) and 279 nm (conjugated ketodienoic acids and α, β, γ, δ-unsaturated ω-oxo acid) simultaneously (Varioskan Flash, ThermoFisher Scientific, USA).

### Determination of kinetic parameters

The kinetic constants of PhLOX2 were measured under standard assay conditions using GLA, AA, EPA and DHA concentrations ranging from 20 to 200 μM; the enzyme concentration was always equal to 12 μM in the reaction mixtures. Total LOX activity was determined spectrophotometrically by measuring the formation of conjugated diene from hydroxyl and hydroperoxy fatty acids at 235 nm. All of the determinations were repeated twice, and the respective kinetic parameters were obtained by plotting the data on a Lineweaver-Burk double-reciprocal graph by OriginPro 8.0. The enzyme activity was expressed in units of enzyme per milligram of total protein, where a unit of enzyme is defined as the absorption increment of 0.001 at 235 nm per minute per milligram of protein at pH 8.0, 20°C.

### Real-time quantitative PCR analysis

The thalli were treated with two different stresses, damage and elicitor. The thalli were damaged by repeated scissor cut and the thalli were treated with 100 μg ml^-1^ oligoagar at 20°C for 1 h. The total RNA of treated samples was extracted using the AxyPre Multisource Total RNA Miniprep Kit (Axygen Bioscientific, Inc., Union City, CA, USA). All samples were ground into powder in liquid nitrogen and homogenized with cell lysis buffer, and the other steps followed the manufacturer’s instructions. Single-stranded cDNA was synthesized from 1 μg of total RNA using Moloney murine leukemia virus reverse transcriptase (Promega Biotech Co., Madison, WI, USA).

The amplification of the PhLOX2 cDNA fragment from *P*. *haitanensis* was performed using special primers designed from available sequences of *P*. *haitanensis* (KC896829). They were Lqs-5′ TCCTTCGTGCTCTTGTTGGTT 3′/Lqa-5′ GCTGCTGTTGTTGGGTTCCT 3′, and the product was 108 bp. Another pair of 18S primers was used to amplify a 153 bp fragment of the 18S as internal reference gene, following as 18Sqs-5′AGTTAGGGGATCGAAGACGA3′/18Sqa 5′CAGCCTTGCGACCATACTC3′. A standard curve was generated for PhLOX2 as well as 18S, and the gene expression levels were normalized by a comparative threshold cycle method. Finally, The relative expression ratio of a target gene can be calculated using the formula 2^-ΔCT^, where ΔCT = (C_T target RNA_-C_T reference RNA_). Data were expressed of three independent experiments.

## Results and Discussion

### Protein sequences and alignments of PhLOX2 from *P*. *haitanensis*

The sequence of *PhLOX2* was found to be 3284 nucleotide long (KC896829). The ORF of this gene has a length of 2700 bp, encoding a protein of 900 amino acids with a molecular weight of 95.7 kDa. The secondary structure of PhLOX2 has been deduced, and the results indicated that this protein should contain 38.93% of *α*-helices, 5.67% of *β*-turns, 17.58% of extended strands, and 37.82% of random coils.

**Fig 1:**
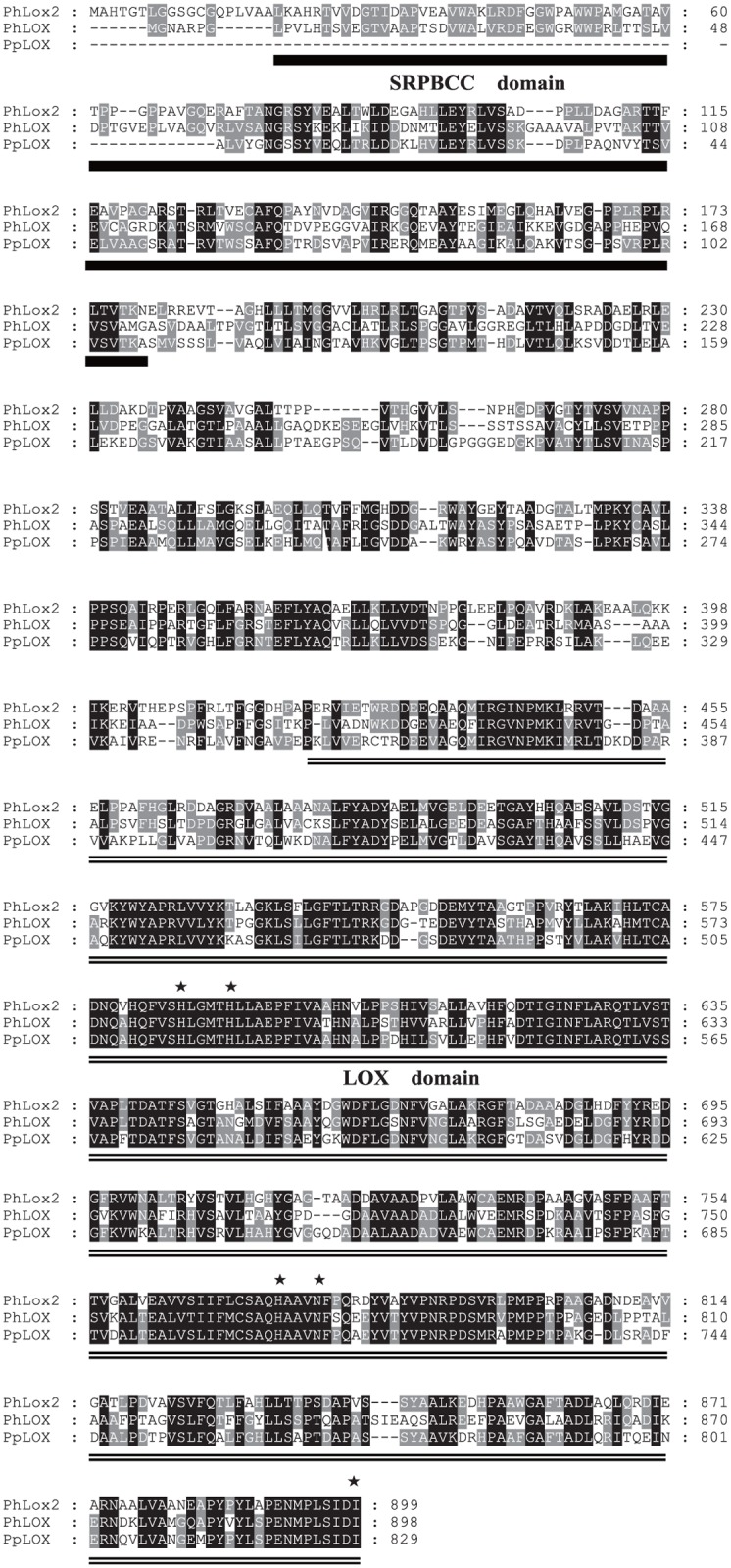
Amino acid sequence alignment. The sequence of PhLOX2 was aligned with the PhLOX protein (AFQ59981) from *P*. *haitanensis* and the PpLOX protein from *P*. *purpurea* (AAA61791) using ClustalX program, and the results were displayed with GeneDoc. Numbers in the right-hand margin refer to amino acid residues. Identical and similar residues are shown on the background as black and gray, respectively. The LOX protein includes SRPBCC (single underline) domain and LOX (double underline) domain. The five essential conserved residues (His-585, His-590, His-774, Asn-778 and Ile-899) in the active site, which are involved in the binding of iron, have been marked with asterisks.

The entire sequence of PhLOX2 showed the highest identity to PpLOX from *P*. *purpurea* (AAA61791) and PhLOX from *P*. *haitanensis* (AFQ59981), whose sequence identity to PhLOX2 was 57% and 50%, respectively. The alignment of amino acid sequence for PhLOX2, PhLOX and PpLOX is shown in Fig. 1. Specifically, a low sequence similarity was found within the N-terminal portion of the protein, which was directed to the SRPBCC (START/RHOalphaC/PITP/Bet v1/CoxG/CalC) superfamily. It has a deep hydrophobic ligand-binding pocket and can bind diverse ligands. Included in this superfamily are the steroidogenic acute regulatory protein (StAR)-related lipid transfer (START) domains of mammalian STARD1-STARD15, and the C-terminal catalytic domains of the alpha oxygenase subunit of Rieske-type non-heme iron aromatic ring-hydroxylating oxygenases (RHOs_alpha_C), as well as the SRPBCC domains of phosphatidylinositol transfer proteins (PITPs). While all the other known LOXs have an N-terminal PLAT domain, which is used to direct the protein to the membrane [12]. The LOX from *P*. *haitanisis* lacks this part of the protein, but its C-terminal domain consisting primarily of α-helices and containing the catalytic sites of the enzyme exhibits similarity to other LOXs. Especially the central histidine-rich region around His-585, His-590, His-774, Asn-778 and Ile-899, which are involved in iron binding within the active site, are highly conserved (Fig. 1, marked by asterisks).

### Phylogenetic analysis of PhLOX2

**Fig 2:**
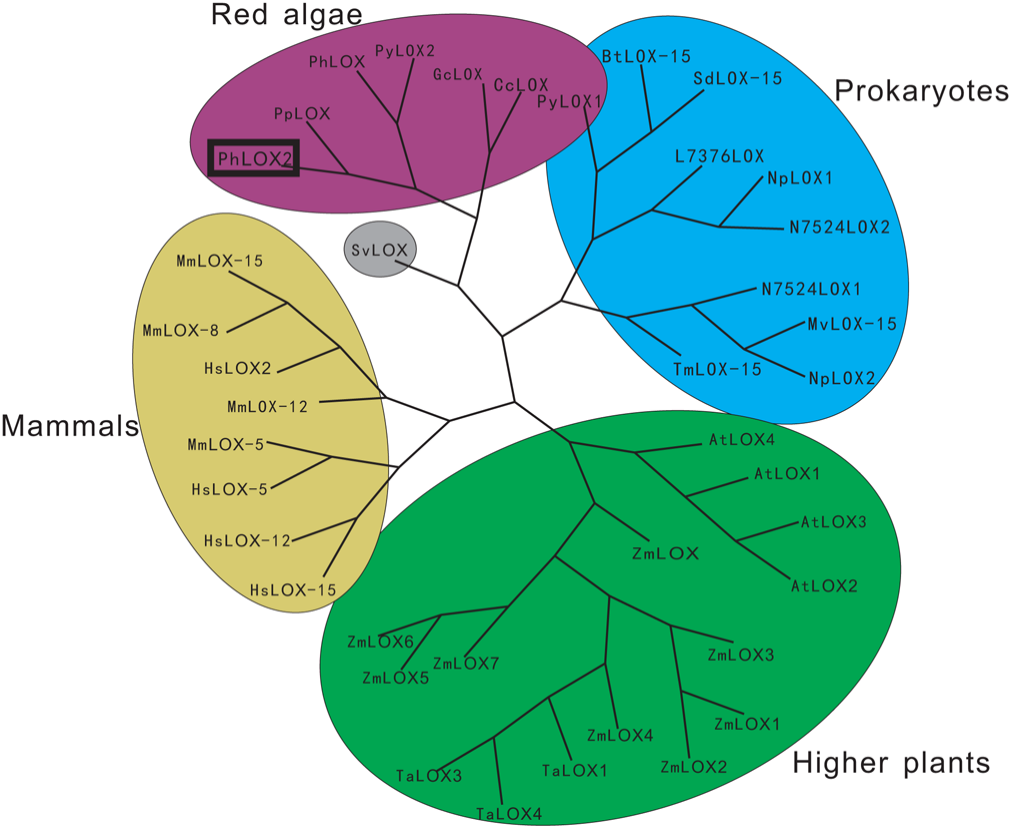
Phylogenetic tree analysis of PhLOX2 and selective LOXs from prokaryotes, plants, mammals, and red algae. The accession numbers of each sequence are described as follows. *Pyropia haitanensis* LOX2 (AGN54275), *Pyropia haitanensis* LOX (AFQ59981), *Pyropia yezeonsis* LOX1–2 (contig 22618 and contig 31643), *Porphyra purpurea* LOX (AAA61791), *Gracilaria chilensis* LOX (AEH16747), *Chondrus crispus* (CHC_T00008739001), *Nostoc punctiforme* LOX1 (YP_001864265), *Nostoc punctiforme* LOX2 (YP_001869751), *Leptolyngbya* sp. PCC 7376 LOX (YP_007070211), *Microcoleus vaginatus* LOX-15 (ZP_08495591), *Nostoc* sp. PCC 7524 LOX1 (YP_007077994), *Nostoc* sp. PCC 7524 LOX2 (YP_007075416), *Thiocapsa marina* LOX-15 (ZP_08768587), *Burkholderia thailandensis* LOX-15 (YP_442874), *Shewanella denitrificans* LOX-15 (YP_562687), *Homo sapiens* LOX-5 (NP_000689), *Shewanella violacea* LOX (YP_003557190), *Homo sapiens* LOX-12 (AAA51533), *Homo sapiens* LOX2 (CAC34521), *Homo sapiens* LOX-15 (NP_001131), *Mus musculus* LOX-12 (NP_033789), *Mus musculus* LOX-5 (AAC37673), *Mus musculus* LOX-8 (AAH15253), *Mus musculus* LOX-15 (NP_033791), *Arabidopsis thaliana* LOX1–4 (CAB56692, AAF97315, NP_564021, NP_177396), *Zea mays* LOX (NP_001105515), *Zea mays* LOX2–7 (AAG61118, DAA50838.1, AEL03787, DAA56391, NP_001105973, NP_001105003, NP_001105975), *Triticum aestivum* LOX1 (ACZ34180), *Triticum aestivum* LOX3–4 (AEB70990, AEB70991). The tree was constructed with Clustalx, TreeView.

The amino acid sequence of the full-length protein was subject to phylogenetic analysis with other known LOX sequences (Fig. 2). The resulting data showed that the sequence of PhLOX2 from *P*. *haitanensis* has low percent identity (<31%) to the mammalian and plant LOXs. The phylogenetic tree of LOX separates the plant, red algae, and animal enzymes and forms several subgroups within each kingdom, indicating that the three branches should have the common ancestral LOX. It is obvious that LOXs from red algae form a distinct group and belong to a separate branch of the tree, as illustrated in Fig. 2. The LOX from a marine bacterium *Shewanella violacea* DSS12 is closely related to this branch, and it contains the same *N*-terminal SRPBCC domain as *Pyropia* and *Porphyra*. Meanwhile, another LOX obtained from *Pyropia yezeonsis* is closely related to that of prokaryotes. It is noteworthy that the LOXs from red algae are more closely related to the ones from prokaryotes than higher plants and mammals, presiding at the early stage of evolution.

### The purification of PhLOX2

**Fig 3:**
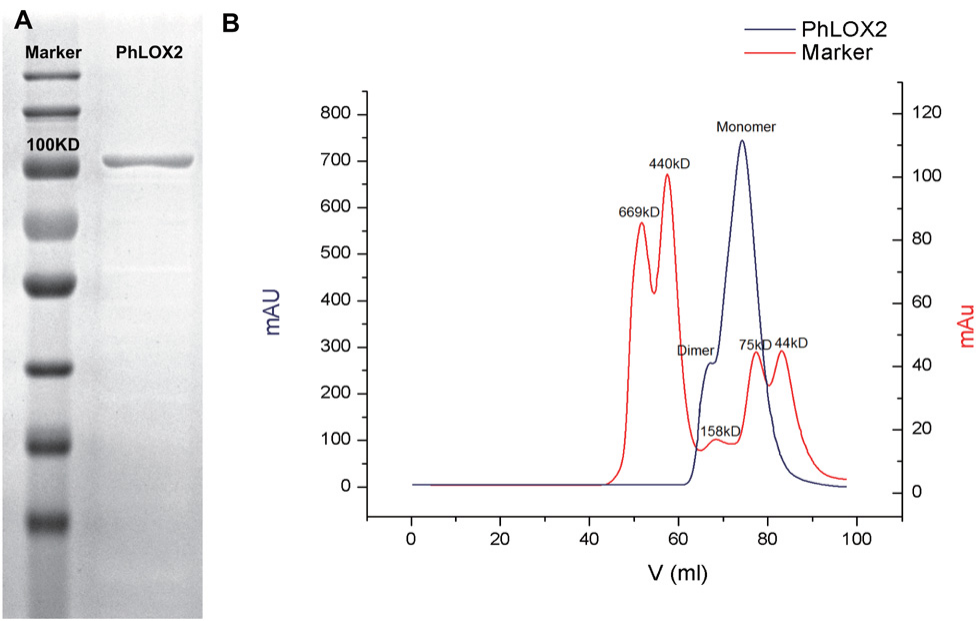
A. SDS-PAGE analysis of purified PhLOX2. B. The size-exclusion chromatography of purified PhLOX2.

PhLOX2 was expressed in-frame with an N-terminal hexa-histidine peptide that enabled the use of affinity chromatography for its purification. The affinity purification step resulted in a final purity of 98% as judged by SDS-PAGE analysis (Fig. 3A) and a yield of up to 83.30 mg protein L^-1^ of culture. The oligomeric state of PhLOX2 was analyzed by size-exclusion chromatography (Fig. 3B). The analysis revealed that this protein showed a MW of about 95 kDa, suggesting that native PhLOX may form a monomer in solution. But a tiny peak of dimer was also observed.

### pH profile of PhLOX2

Generally, the conjugated diene fragment in hydroperoxy and hydroxy fatty acids can exhibit a maximum absorption peak at 235 nm, whereas the conjugated ketodienoic acids and α, β, γ, δ-unsaturated ω-oxo acids often show their maximum absorption peaks at 279 nm. In this work, EPA substrate was allowed to react with the enzyme, and the UV absorption of the resulting products was subsequently measured, affording a strong peak at 235 nm. However, the absorption peak at 279 nm was absent. These results suggest that hydroperoxy or hydroxy fatty acids should be the products generated. Other substrates employed in this transformation also afforded similar results, except that when LA and ALA were allowed to react with the enzyme, no UV absorbance was observed.

**Fig 4:**
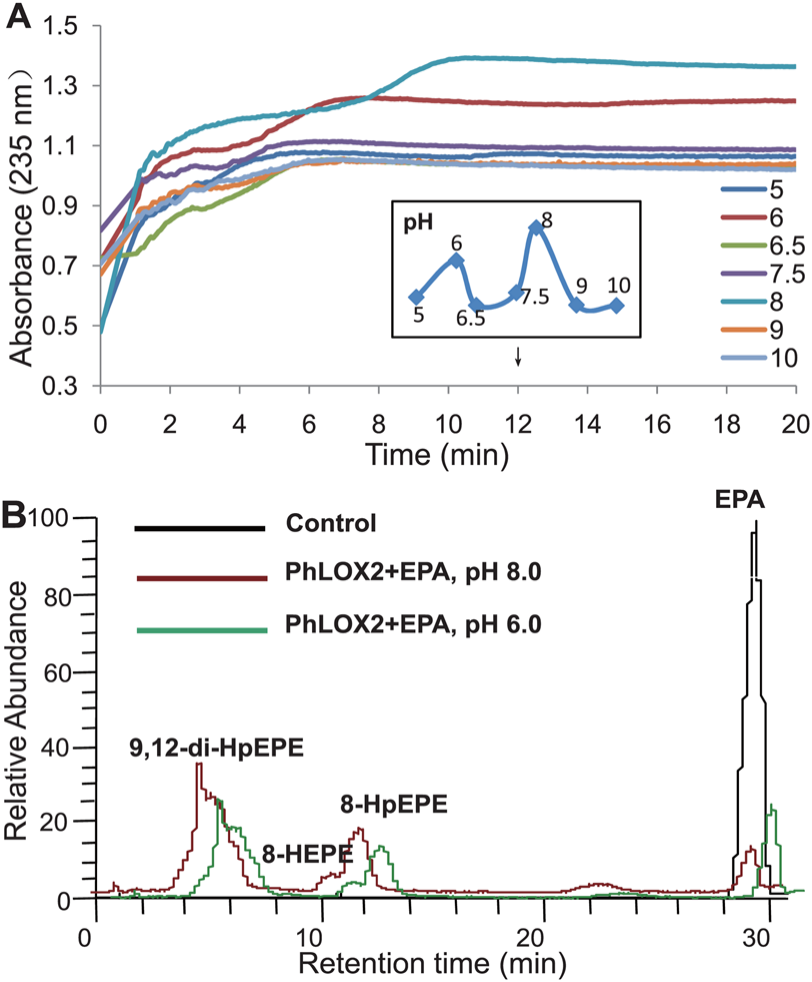
pH optimization for the PhLOX2 activity array (235nm). A, The activity array for PhLOX2 with EPA. UV absorption was measured at 235 nm, which is the absorption wavelength for hydro(pero)xidized fatty acids. B, The alteration of PhLOX2 reaction products catalyzed under different pH.

Subsequently, the “optimal” pH for PhLOX2 was determined, primarily by measuring the UV absorbance of substrates during the course of conversion. Fig. 4A showed the UV absorbance of EPA that was being converted in the presence of PhLOX2 catalyst, in which the highest activity for PhLOX2 was found at pH 8.0, and the catalytic reaction turned out to be fairly fast under this condition. The activity dramatically decreased when pH was above 8.0. When the pH is lower than 8.0, the enzyme is also quite active; as a matter of fact, we observed that the absorbance showed two peaks at pH 6.0 and pH 8.0, respectively at 12 min. Because all products containing -OH or -OOH functional groups exhibit an UV absorption peak at 235 nm, when the products are composed of a mixture of compounds with -OH and -OOH functional groups, the sole measurement of UV absorption at 235 nm may not reflect the pH effect on the individual enzyme activity that is required for the formation of different products. For PhLOX2, these results clearly suggest that the reaction pathways and factors affecting enzyme activity may be fairly complicated. Therefore, we analyzed the products generated from the catalytic reactions of EPA under pH 6.0 and pH 8.0 by HPLC. Fig. 4B revealed that no difference is evident between the products generated under two pH conditions. The three main oxylipins including 8-HpEPE, 8-hydroxyeicosapentaenoic acid (HEPE) and 9, 12-diHpEPE have been observed in these two spectra, but the height of each peak seems to be different. The product contents under pH 8.0 were higher than the ones under pH 6, indicating that pH should not affect the catalytic mode, but the activity of PhLOX2. However, the intrinsic reason why the absorbance at 235 nm decreased between pH 6.5–7.5 is still not clear.

### Substrate specificity and kinetic analysis of PhLOX2

**Fig 5:**
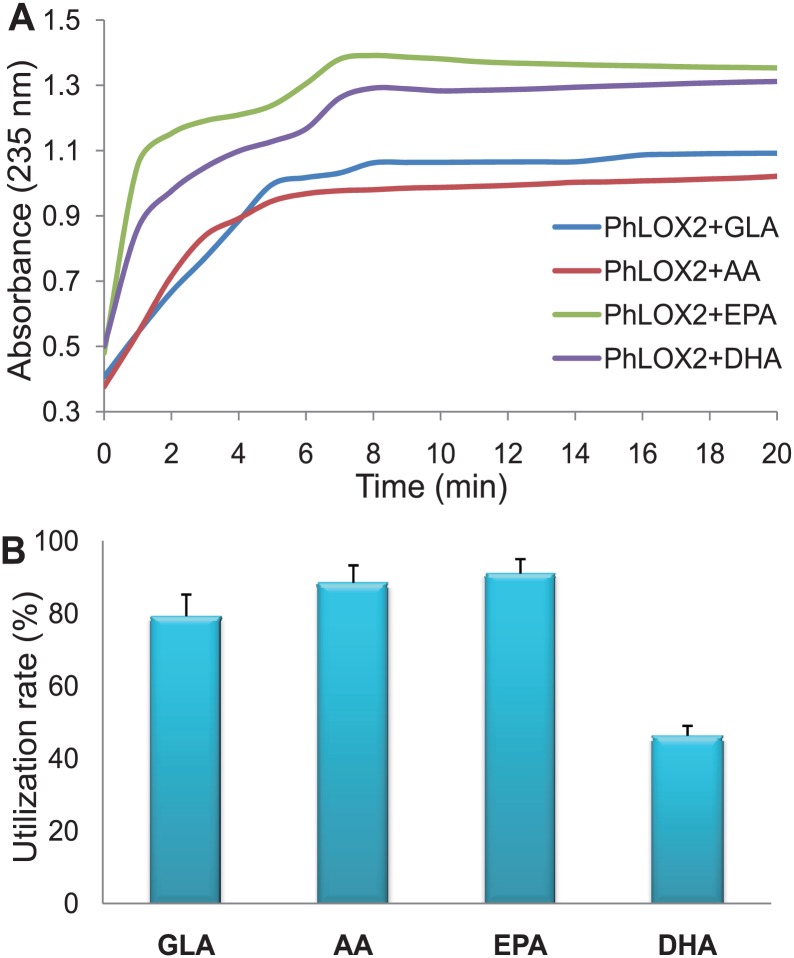
PhLOX2 activity array with different substrates at pH = 8.0 (235nm). A, Activity array for PhLOX2 with different substrates. UV absorption was measured at 235nm, which is the absorption wavelength for hydro(pero)xidized fatty acids. B, Catalytic selectivity of PhLOX2 for PUFA substrates. The mixture of four fatty acids (GLA, AA, EPA, and DHA, 100 μM solution) was allowed to react with PhLOX2 for 30 min. The utilization rate of each substrate = (content of each substrate before reaction)-(Content of each substrate after reaction)/(content of each substrate before reaction)×100%.

At pH 8.0, four different substrates were allowed to react with PhLOX2, and the resulting products were analyzed subsequently. For substrates EPA and DHA, it appeared that two reaction stages were present, and the reaction rate for the first stage was fairly high (Fig. 5A). In fact, the reaction reached its first high point in 1 min, and the maximum absorption was obtained around 7 min. For substrates AA and GLA, the UV absorption seemed to slowly increase at the beginning of the reaction, and the latter achieved its maximum absorption at 8 min. Therefore, we conclude that PhLOX2 can utilize EPA and DHA faster and more efficiently. We also determined the optimal substrate of PhLOX2 by kinetic analysis. The kinetic characterization of PhLOX2 was carried out with four substrates, GLA, AA, EPA and DHA. The kinetic constants K_m_ and K_cat_ are reported in Table 1. The lowest K_m_ value was recorded with EPA, and this indicates that PhLOX2 has the highest affinity for EPA and is particularly active towards EPA. The K_m_ value was the highest when GLA was used as the substrate. The K_cat_/K_m_ values show that EPA was most efficiently catalyzed by PhLOX2, and GLA showed the lowest K_cat_/K_m_ value. It should be noted that the K_cat_ values for other substrates differed significantly with that of K_m_. Although the K_m_ of DHA was lower than AA and GLA, it showed the lowest K_cat_. Considering that the hydroperoxy products formed are further metabolized into hydroxy-, dihydroperoxy fatty acids, the composition and proportion in the products may different remarkably. This fact might be the molecular basis for the different kinetic parameters. These data support that EPA was the optimal substrate for PhLOX2; in fact, GLA was a poor substrate.

**Table 1:** Kinetic characterization of PhLOX2.

Substrates	K_m_ (μM)	K_cat_ (s^-1^)	K_cat_/K_m_ (s^-1^μM^-1^)
GLA	150.64	22.98	0.152
AA	114.17	47.47	0.416
EPA	54.09	62.5	1.155
DHA	91.59	12.62	0.138

Furthermore, we aimed to determine the preferred substrates for PhLOX2 by incubating the purified protein with a mixture of GLA, AA, EPA and DHA. The disappearance of PUFAs by incubation with the purified protein in comparison with the control was measured by LC-MS. Notably, the results showed that PhLOX2 can catalyze C18, C20 and C22 fatty acids. For C20 fatty acids, EPA and AA turned out to be the main substrates converted (90.97% and 88.43%, respectively). In contrast, the conversion of GLA and DHA was much less, which was 79.21% and 46.26%, respectively (Fig. 5B). Such preference in substrate utilization is consistent with the results obtained from other red algae. For red algae, C20 PUFAs (AA and EPA) and C18 PUFAs (LA and ALA) have been employed as substrates, and the most prominent theme in red algal oxylipin biosynthesis seems to be the metabolism of C-20 PUFAs [4]. In addition, these results are in agreement with the previous findings involving the response of *P*. *haitanensis* to agaro-oligosaccharides, in which AA and EPA are the main precursors for the structurally diverse, unsaturated aldehydes and alcohols [11]. By comparing these results with the data illustrated in Fig. 5A and the kinetic characterization of PhLOX2, we found that EPA exhibited the highest degree of utilization and conversion rate. As shown in Fig. 5B, AA also manifested high degree of utilization; however, only 46.26% DHA was consumed. Based on the absorption at 235 nm as shown in Fig. 5A, the consumption of DHA was extremely fast and the 235 nm UV absorption reached a fairly high value, whereas that of AA was low. This discrepancy indicated that DHA is not the preferred substrate for PhLOX2 in the presence of other substrates, and the catalytic conversion of DHA may only high efficiently proceed when it is used alone. Alternatively, PhLOX2 may catalyze the conversion of AA and DHA to produce mono-hydroperoxy, di-hydroperoxy and hydroxyl compounds at different ratio. Because the absorptions of those products at 235 nm are different, even DHA was consumed less than 50%, the resulting absorbance at this wavelength may still fairly strong.

### Flexibility of PhLOX2 catalytic site

**Fig 6:**
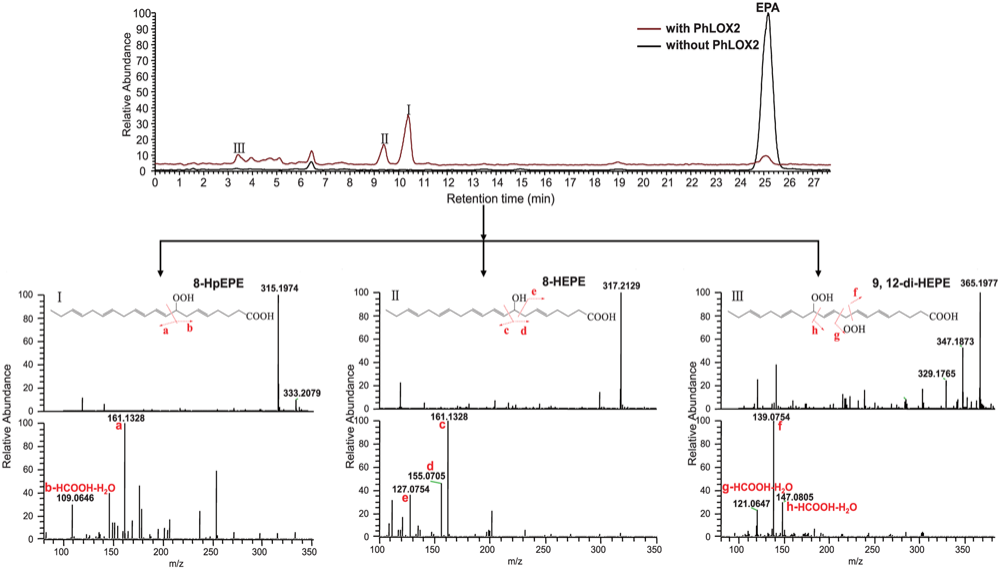
LC-MS analysis for the catalytic reaction of EPA with PhLOX2. (I), (II) and (III) showed the mass spectra and the corresponding MS/MS fragmentation scheme of 8-HpEPE, 8-HEPE and 9,12-diHpEPA, respectively.

The catalytic activity of PhLOX2 against LA, ALA, GLA, AA, EPA and DHA has been closely examined with the assistance of LC and high resolution mass spectrometry analyses, as shown in Table 2. We found that PhLOX2 showed no activity to LA and ALA. Fig. 6 listed the LC and high resolution MS/MS spectra of different types of products (mono-, di-hydroperoxy and hydroxyl compounds) from the PhLOX2 catalyzed reactions of EPA. In the MS spectrum, [M-H]^-^ ions at m/z 315.1974, 317.2129 and 365.1977 for 8-HpEPE, 8-HEPE and 9, 12-diHpEPE were all observed. Besides, the proposed fragmentation pathways were also included.

**Table 2:** Major products from the reaction of PhLOX2 with different fatty acids and the molecular ions identified ([M-H]^-^).

Substrates	Products	MS (m/z)	MS/MS (m/z)
**GLA (γ-18:3)**	**6-HpOTE**	291.1968, 309.2078	83.0488, 163.1484, 189.1274
	**9-HpOTE**	291.1966, 309.2076	123.0804, 151.1118, 165.1278
	**13-HpOTE**	291.1967, 309.2077	113.0960, 139.1119, 165.1278, 193.1225
	**9-HOTE**	293.2122	123.0804, 141.0911, 151.1119, 169.0863
**AA (20:4)**	**8-HpETE**	317.2131, 335.2236	109.0647, 111.0439, 163.1484, 205.1595
	**8-HETE**	319.2287	111.0805, 127.0753, 155.0705, 163.1486
	**9,12-di-HpETE**	331.1922, 349.2028, 367.2133	121.0647, 139.0754, 147.0805, 183.0657
**EPA (20:5)**	**8-HpEPE**	315.1974, 333.2079	109.0646, 161.1328, 203.1436
	**8-HEPE**	317.2129	111.0803, 127.0754, 155.0705, 161.1328
	**9,12-di-HpEPE**	329.1765, 347.1873, 365.1977	121.0648, 139.0754, 147.0805, 183.0657
**DHA (22:6)**	**7-HpDHE**	341.2133, 359.2238	109.0648, 121.0649, 147.1171, 161.1330, 173.1327, 201.1645, 243.1753
	**10-HpDHE**	341.2132, 359.2240	121.1013, 135.0806, 151.0754, 161.1329, 189.1270
	**7-HDHE**	343.2287	201.1653, 141.0547, 123.0806, 113.0595
	**10-HDHE**	343.2288	153.0913, 161.1329, 181.0869, 121.0648
	**7,14-di-HpDHE**	355.1925, 373.2031, 391.2142	175.1122, 137.0962, 135.0805, 121.0648, 95.0489
	**4,20-di-HpDHE**	355.1924, 373.2030, 391.2138	109.0647, 115.0390, 147.1167, 81.0332

Specifically, results obtained from the *in vitro* biochemical analysis revealed that the PhLOX2 protein possesses broad substrate tolerance, and it can metabolize fatty acids with chain lengths of C20, C22 and also γ-C18:3, affording hydroperoxy and hydroxyl fatty acids containing different positional isomers. This is in accordance with the presence of a large number of structurally different oxylipins found in other red algae [13]. These diverse oxylipins can be generated by a few catalytic enzymes, which in a way explaining how sophisticated functions can be achieved in red algae with a simple genome. In contrast, mammals and higher plants only use C20 or C18 fatty acids as reaction substrates, respectively. Furthermore, C18 fatty acids used by higher plant LOXs and PhLOX2 are different. PhLOX2 shows selectivity towards C18 fatty acids and only catalyzes the conversion of *γ*-linolenic acid, but most of higher plants prefer to convert *α*-LA, only the LOX enzyme in *Physcomitrella patens* has been reported to catalyze transformation of *γ*-linolenic acid substrate to form the major product 10-γHpOTE [2].

**Fig 7:**
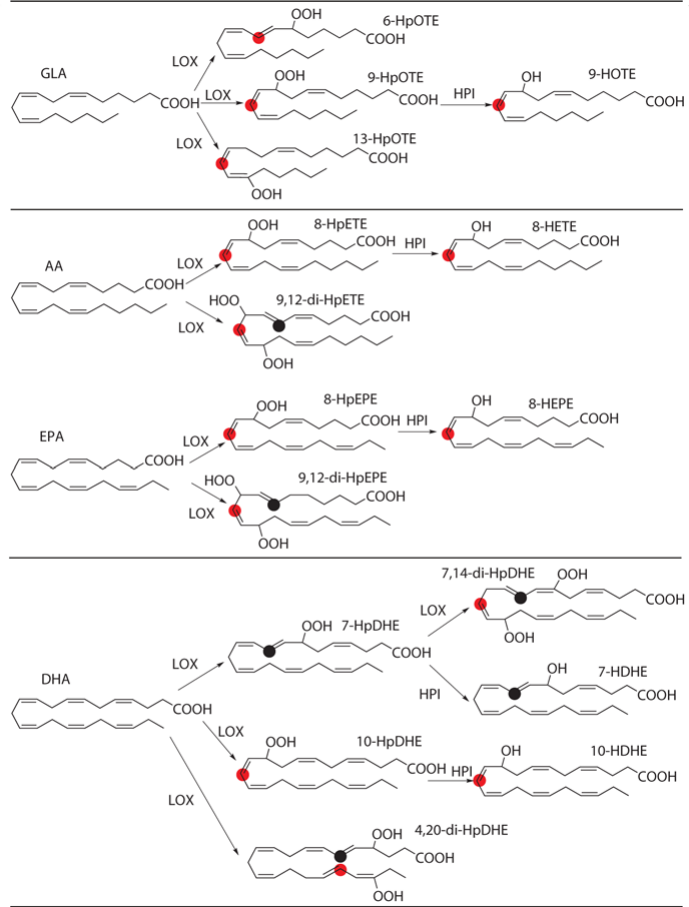
PhLOX2 catalyzed reactions. Pathways for PhLOX2 catalyzed reactions using four substrates. GLA, AA, EPA, and DHA were employed as reaction substrates, and the resulting products were monitored with LC-MS analysis. The deduced reaction pathways are illustrated in the diagram. Red and black dots represent the reaction site of the radical oxygenation reaction.

Fig. 7 shows the PhLOX2-catalyzed pathways based on the different types of hydroperoxy and hydroxyl compounds detected. Particularly, the structural types for the products obtained from PhLOX2 catalyzed reactions are fairly diverse, including mono-hydroperoxy, di-hydroperoxy and hydroxyl products, revealing it to be an unconventional LOX with no strictly positional specificities but some catalytic rules. Multiple catalytic sites are available, such as 6-/9-/13-lipoxygenase against GLA; 8-/9-/12-lipoxygenase against AA and EPA; and 4-/7-/10-/14-/20-lipoxygenase against DHA. Notably, GLA is mainly converted to 9-hydroxy-7,9,12- octadecatrienoic acid (9-HOTE), which has been identified as a 9-LOX product. Meanwhile, the 6-LOX activity (6-HpOTE) against GLA has also been detected, yet it is not evident in other higher plants and mammals. These activities might suggest that substrate binding in the active site of PhLOX2 was more flexible in comparison with other LOXs. Although both LOXs in higher plants and PhLOX2 can utilize C18 fatty acids as substrates to produce 9- or 13-hydroperoxides, the LOX enzymes in higher plants usually use α-linolenic acid as the substrate to form the biologically active oxylipins such as Jasmonic acid. In contrast, the PhLOX2 enzyme can only catalyze the reaction of γ-linolenic acid in *P*. *haitanensis*, and the function of resulting downstream products is still unclear. When C20 PUFAs were employed, the 8-LOX activity has been observed in both *P*. *haitanensis* and animals. 8-LOX is one of the several LOXs expressed in mouse epidermis, and its occurrence is barely detectable in normal skin cells, whereas it is highly expressed in phorbol ester-treated hyperproliferative epidermis of various mouse strains [14]. In corals, it was suggested that prostaglandin-like molecules could be produced from 8-hydroperoxy eicosatetraenoic acid (HpETE) by enzyme mediated reactions [15]. In addition, it is possible that the synthesis of prostaglandins in red algae *Glacilaria verrucosa* [16] and *Glacilaria lichenoids* [17] could proceed through a similar pathway, in which 8-HPETE is involved in the biosynthesis. As shown in Fig. 7, both 8-HpEPE and 8-HpETE have been detected in *P*. *haitanensis*, strongly suggesting that red algae should contain a functional 8-LOX system. In *P*. *haitanensis*, however, no prostaglandins were detected or reported. Meanwhile, rather than the commonly expected hydroperoxy compounds, 8-LOX in PhLOX2 can further catalyze the conversion of 8-HpEPE and 8-HpETE to generate hydroxyl products (8-hydroxy eicosatetraenoic acid (HETE) and 8-HEPE), as shown in Fig. 7. Therefore, the exact function of 8-LOX in this alga is still unclear. Further analysis revealed the presence of several unusual hydroperoxy products when DHA was used as substrate, such as 7-HpDHE and 10-HpDHE which are not reported in the literatures. The formation of these mono-hydroperoxy products suggested that free radicals should be initiated at two specific locations on the fatty acid backbone chain, specifically, the carbon atom between the first double bond and the second double bond, or the one between the second double bond and the third double bond, as marked by red dots in Fig. 7. However, in DHA, the carbon atom between the third and the fourth double bond was also attached to form 10-HpDHE, plus, the double bonds are closer to the carboxylic acid in DHA than other PUFAs, therefore, the oxygenation position may be closer to the front, depending on the substrate inserting position in the active site pocket. Furthermore, we noticed that except the 13-HpOTE of GLA, the -OOH group of other mono-hydroperoxy compounds are all in the vicinity of carboxylic acids at (n-2) position. The PhLOX2 enzyme has demonstrated its signature characteristics, which is the requirement of triple ethylenic bonds, consistent with the fact that PhLOX2 did not shown activity to LA. The distance from ethylenic bond to carboxylic group could affect the position of hydrogen abstraction in substrates, therefore, because PhLOX2 showed activity to γ-linolenic acid, but not to α-linolenic acid, we conclude that although the substrate binding in the active site of PhLOX2 could be flexible, certain structural requirement for substrates are still needed.

### Two site oxidation activity of PhLOX2

**Fig 8:**
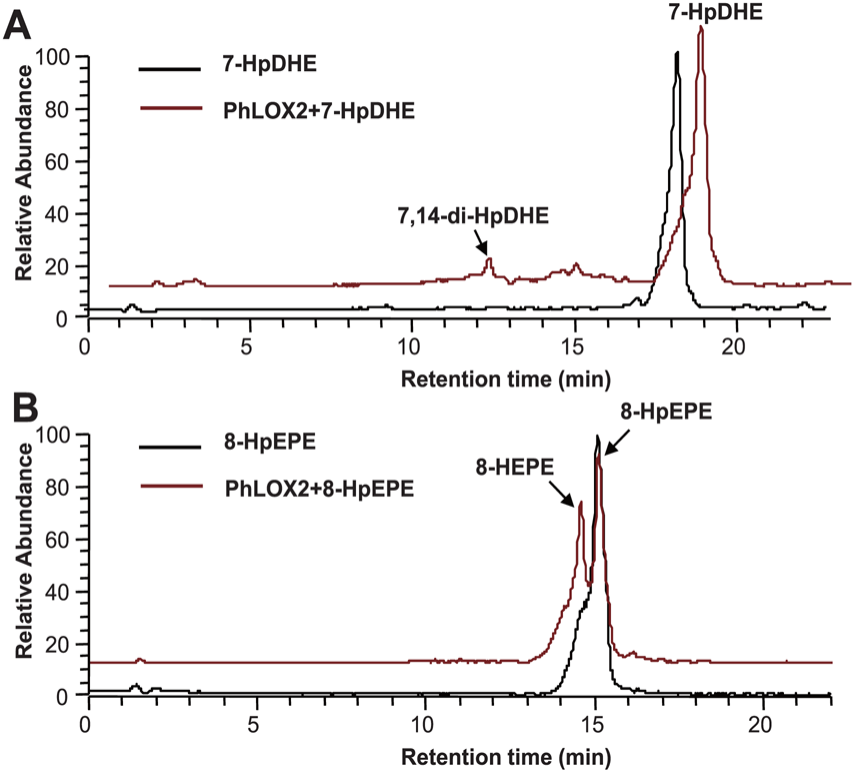
Product specificity of PhLOX2 on hydroperoxide intermediates. A, 7-HpDHE as substrate. B, 8-HpEPE as substrate.

Besides mono-hydroperoxy products, certain di-hydroperoxy products can also be generated from the PhLOX2 catalyzed reactions, such as 9,12-di-HpETE from AA; 9,12-di-HpEPE from EPA; 7,14-di-HpDHE and 4,20-di-HpDHE from DHA (Table 2, Fig. 7). Such “double di-oxygenation” properties have also been reported in a few LOXs, including the soy LOX1, wheat seed LOX, and rice seed LOX-2, which can be employed to convert AA to a mixture of 5,15- and 8,15-diHpETE [18]. It appears that four ethylenic bonds are required for the formation of di-hydroperoxy products from polyunsaturated fatty acids; therefore, no di-oxygenation product was formed when GLA was employed as the substrate. Based on the reaction site of hydroperoxylation, it was hard to summarize rules. The generation of di-hydroperoxy products may not go through the two-step pathway, and some researchers have proposed a forward-inverse subsequent entry theory, in which the production of di-hydroperoxides is mediated by soybean LOX-1 capable of catalyzing 15, 5, and 8-LOX reactions. In this theory, the initial oxygenation reaction to form 15-HpETE is followed by its reverse orientation (“flip”), and then by 5 or 8 oxygenation to produce 5,15-di-HpETE or 8,15-di-HpETE. Consequently, 15-HpETE can be detected in the products obtained [19]. However, in our work, no 9- and 12- mono-hydroperoxy compounds from AA and EPA, or 4- mono-hydroperoxide from DHA were detected, instead, only 7-HpDHE was found, which may be the reactive precursor for 7,14-diHpDHE. In order to demonstrate this hypothesis, we purified 7-HpDHE and used it as substrate. We found 7, 14-diHpDHE was formed (Fig. 8A), which indicated that di-hydroperoxy products should be derived from mono-hydroperoxide by enzymatic reaction. The missing of mono-hydroperoxide precursor for other di-hydroperoxy products could be due to fact that the second reaction step is unusually fast, which could prevent the detection of the products from the first reaction step. To verify this hypothesis, structural modeling of the enzyme/substrate complex needs to be performed. For AA and EPA, the oxygenation positions are the same, and hydrogen abstraction occurs at carbon atoms C-7 and C-10, plus, molecular oxygens can insert into position (n+2), ultimately forming the di-hydroperoxy products 9,12-diHpETE and 9,12-diHpEPE.

### Hydroperoxidase activity of PhLOX2

Other than LOX activity, PhLOX2 also possesses hydroperoxidase activity, by which hydroperoxy PUFAs can be converted into their hydroxide PUFA counterparts, thus it should be deemed as a multifunction enzyme. Particularly, when PhLOX2 was incubated with GLA, the formation of 9-hydroxyoctadecatrienoic acid (HOTE) has been identified, as illustrated in Fig. 7. Subsequently, different substrates were employed to explore its enzymatic activity and it was found that the C18, C20 and C22 PUFAs can be hydroxylated, affording 9-HOTE, 8-HETE, 8-HEPE, 7-hydroxy docosahexaenoic acid (HDHE) and 10-HDHE as products. In order to verify the hydroxyl fatty acids products were derived from hydroperoxy precursors by enzymatic reaction, we also purified one intermediate product 8-HpEPE and used it as a substrate. The LC chromatogram displayed the emerge of 8-HEPE (Fig. 8B), indicating the two step reactions were all catalyzed by PhLOX2.

Notably, the hydroxy products are present in both higher plants and mammals. Specifically, 9-HOTE and 13-HOTE are found in higher plants, and 5-HETE, 8-HETE, 12-HETE as well as 15-HETE are evident in mammals [15]. Furthermore, such compounds like 8-HETE also exist in coral or red algae [2]. In red algae, the formation of hydroxy products seems to be ubiquitous. For instance, 8-HETE, 5-HETE and 5-HEPE have been identified as metabolites in Rhodophyta *Agardhiella subulata* [20] and *Rhodymenia pertusa* [21]. In a recent study, after methyl jasmonate (MeJA) treatment, cell-free extracts of *C*. *crispus* gametophytes were incubated with C18 and C20 PUFAs, and the resulting products contained 11-hydroxy octadecaenoic acid (HODE), 11-HOTE, 13-HETE and 13-HEPE [16], which suggested that these hydroxyl products in red algae may serve as chemical defense signal carrier.

The origin of hydroxy compounds varies, but the most common one seems to be the reduction of hydroperoxy intermediates [15]. Moreover, some products are generated via enzyme-catalyzed processes, in which certain hydroperoxy intermediates can be converted to mono-hydroxy products in the presence of peroxidase. For instance, 9-HETE in *Polyneura latissima* has been postulated to be originated from the peroxidase activity [22]. It is known that purified prostaglandin hydroperoxidase from bovine vesicular gland microsomes can convert prostaglandin G1 to prostaglandin H1 [23]. Additionally, di-hydroxy products can be obtained with the assistance of linoeate diol synthase (LDS) or PpOA (a putative oxylipin producing oxygenase). Specifically, it was reported that LA can be converted to 7,8-di-hydroxy octadecaenoic acid (diHODE) in *Gaeumannomyces graminis* by LDS [24–26], and the generation of 5,8-diHODE from LA in *Aspergillus nidulans* has been achieved by PpOA. Notably, these enzymes all possess single catalytic activity, but the enzyme PhLOX2 appears to be a dual function molecule, because it serves as both lipoxygenase and hydroperoxidase. In 1997, Koljak et al. reported a peroxidase-lipoxygenase fusion protein from coral *Plexaura homomalla*, and it can convert arachidonic acids to allene oxides [27], which is the first example of LOX exhibiting peroxidase activity. In addition, catalase like allene oxide synthase-LOX fusion proteins has been disclosed in Cyanobacterium *Acaryochloris marina* [28], *Anabaena PCC7120* [29] and coral *P*. *homomalla* [30, 31]. However, these dual function proteins are fundamentally different from PhLOX2, because they are essentially constructed by fusing two functional domains together. In contrast, PhLOX2 is a single domain protein exhibiting both hydroperoxidase and lipoxygenase functions. Besides, the products formed are also not the same, because the fusion proteins are generally involved in the formation of allene oxides, and PhLOX2 serves as the catalyst for the production of mono-hydroxy fatty acids. Moreover, peroxidase, hydroperoxidases, catalase-like allene oxide synthase and LDS are all heme-dependent enzymes, whereas PhLOX2 is not, even though it has hydroperoxidase activity. Interestingly, Brodhun et al. found that a fungal FoxLOX, a non-heme iron lipoxygenase in *Fusarium oxysporum*, also exhibits multifunctional activity by oxidizing C18-polyunsaturated fatty acids to 13S-hydroperoxy derivatives. With α-linolenic acid as the substrate, the hydroperoxy derivatives formed by FoxLOX are further converted to dihydroxy-, keto-, and epoxy alcohol derivatives [32], which were similar to PhLOX2.

The intrinsic mechanism involved in the enzyme-catalyzed hydroxylation is not clear yet, but meaningful information still can be gathered by comparing the hydroxy products obtained. Because the products derived from LA and ALA were not evident, it is unlikely that PhLOX2 can catalyze the conversion of C18 PUFAs, but it should show decent activity for the conversion of C20 or C22 PUFAs. The selection of hydroxylation site seems to follow certain rules, and the hydrogen residing on the carbon atom between the second and third double bond next to the carbonyl acid motif should be the one abstracted, such as C-11 in GLA, C-10 in AA or EPA, and C-9 in DHA. Molecular oxygen is introduced at the position (n-2), leading to the formation of 9-HOTE for GLA, 8-HETE for AA, 8-HEPE for EPA and 7-HDHE for DHA. Similar to the mono-hydroxyperoxide of DHA (10-HpDHE), we have observed the presence of 10-HDHE, which does not obey the rule mentioned above. Overall, these results could be correlated with the structure activity relationship between enzyme and substrates.

### Expression of *PhLOX2* under different stresses

**Fig 9:**
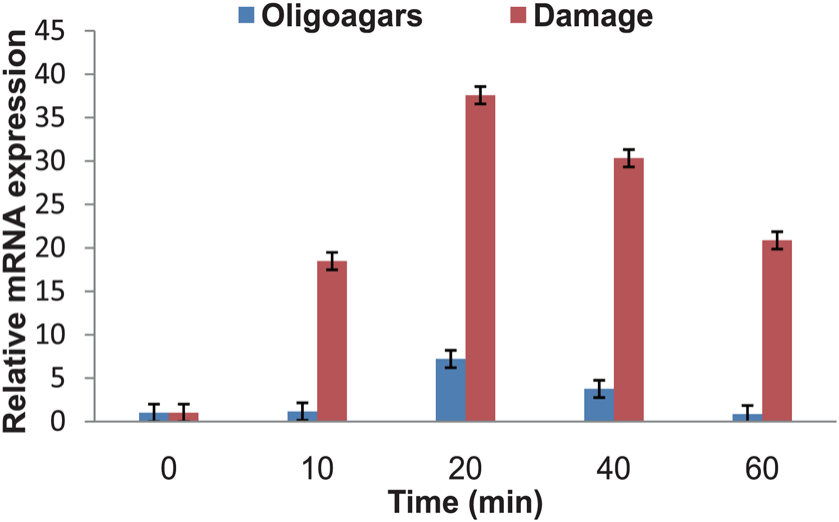
Expression level of *PhLOX2* under different stresses. The thalli were treated with two different stresses, damage and elicitor. The thalli were damaged by scissor cut repeatedly and the thalli were treated with 100 μg ml^-1^ oligoagars at 20°C for 1 h. The mRNA of treated samples was extracted and the expression of *PhLOX2* was analyzed by QRT-PCR.

As intertidal algae, Pyropia experience alternating aquatic and terrestrial environments and undergo dynamic environmental stresses [33]. To survive in such a variable environment, Pyropia develop strategies to respond to stresses. Many eukaryotic algae have been found to develop oxylipin mechanisms ubiquitously to defend themselves against both abiotic and biotic stresses [34]. In the present study, as the pivotal enzyme to synthesize oxylipins, the increased *PhLOX2* expression was observed following oligoagars stimulation and damage treatment for 1 h (Fig. 9), but the damage induced more striking increase of *PhLOX2* expression. In 10 min, the level increased to 18.48-fold, and it reached the maximum expression of 37.59-fold at 20 min. Oligoagars also induced the expression of *PhLOXs*. At 20 min, the level of *PhLOX2* was increased by 7.2-fold. In the previous research on the response of *P*. *haitanensis* to desiccation, we found that expression of *PhLOX2* showed remarkable gene expression increase after 4 h desiccation, an 8.7-fold increase comparing with that of the control [35]. Therefore, we suggest that PhLOX2 could be part of the *P*. *haitanensis* defense system.

## Conclusion

In summary, we have disclosed a novel multifunctional enzyme PhLOX2, which possesses flexible catalytic position characteristics and exhibits both lipoxygenase and hydroperoxidase activities. Particularly, four fatty acids, including the C20 PUFAs as the main substrates, have been employed in the PhLOX2 catalyzed reactions, affording 16 hydroperoxy fatty acids and hydroxy fatty acids. Aside from common products found in higher plants and mammals, a few unique oxylipin analogs were produced by this transformation. Notably, the PhLOX2 enzyme can only tolerate unsaturated fatty acids containing at least three double bonds. At this moment, the reaction mechanism involving LOX protein is still not clear, and further efforts to elucidate the mechanism by crystal structure analysis are currently in progress.
